# Development of a Questionnaire for Measuring Employees’ Perception of Selection, Optimisation and Compensation at the Leadership, Group and Individual Levels

**DOI:** 10.3390/ijerph18126475

**Published:** 2021-06-15

**Authors:** Annette Meng, Iben L. Karlsen, Vilhelm Borg, Thomas Clausen

**Affiliations:** National Research Center for the Working Environment, Lersø Parkallé 105, 2100 Copenhagen, Denmark; ika@nfa.dk (I.L.K.); vbo@nfa.dk (V.B.); tcl@nfa.dk (T.C.)

**Keywords:** SOC, developmental psychology, sustainable work, ageing at work, organisational psychology

## Abstract

This study is the first to develop a questionnaire to measure employees’ perceptions of the use of the action strategies selection, optimisation, and compensation (SOC) beyond the individual level, which has so far been lacking in research and practice. The study thus contributes an important tool for research into the role of SOC strategies at the leadership, group, and individual levels for long and healthy working lives. It may also be used by practitioners as a tool to provide input when developing targeted interventions to support long and healthy working lives. The development of the questionnaire was based on SOC theory, qualitative and cognitive interviews, and existing SOC questionnaires. The validity and reliability of the questionnaire were tested on data from a cross-sectional survey with responses from 785 nurses and 244 dairy workers. Results from confirmatory factor analyses supported the theoretically expected nine-factor structure of the questionnaire in both study populations (nurses and dairy workers). Furthermore, the results largely supported the criterion validity and internal reliability of the scales in the questionnaire. Nevertheless, further validation across additional occupational groups is needed.

## 1. Introduction

As the workforce ages, the need to create workplaces that support long and healthy work lives increases. The SOC model is a life span model that theorises how people can age successfully through the use of selection, optimisation, and compensation strategies [1], and it has been applied when studying successful ageing at work, e.g., [2,3,4]. The use of selection, optimisation, and compensation (SOC) strategies [1] has been found to be positively associated with important work-related outcomes such as workability [5,6,7,8,9,10], wellbeing [11,12,13,14], work engagement [15,16,17], job performance [18,19], and job satisfaction [20,21]. The use of SOC strategies may thus support a long and healthy work life. 

So far, only questionnaires measuring individual employee’s use of SOC strategies have been developed. However, sustainable employability [22], or the achievement of long and healthy work lives, is complex and depends on many factors beyond the individual. Explicitly exploring collective strategies used by the group of colleagues or the management may potentially provide new and important insights into how workplaces can support sustainable work lives for the employees. We therefore developed a questionnaire to measure employees’ perception of the use of SOC strategies at the leadership, group, and individual levels. The aim of this study was to test the validity and reliability of the questionnaire. 

*Selection* refers to the setting and prioritisation of goals as a response to a reduction in resources. A distinction is made between *loss-based selection* referring to the involuntary abandonment of goals, and *elective selection* referring to the voluntary selection or prioritisation of goals based on personal motives and preferences. *Optimisation* refers to the investment of means and allocation of resources to reach the goal. Lastly, *Compensation* refers to the use of alternative means or external resources to reach the goal [1]. The SOC model describes strategies people use throughout their lifespan to maximise gains and minimise losses [1]. SOC strategies may be applied in response to a reduction in resources caused by developmental changes, as well as external changes, such as organisational changes, throughout the career. Although some studies have found that older workers benefit more from the use of SOC strategies than younger workers [2,10,23], other studies have found that workers across all age groups benefit from the use of SOC strategies [13,21]. von Bonsdorff [6] even found that the strength of the association between the use of SOC strategies and work ability was stronger among younger than older nurses. These findings highlight the potential benefits of using SOC strategies throughout the entire career. Therefore, we developed a questionnaire that measures the use of SOC as a response to limited resources or to maintain resources at all stages of the career but does not primarily target older workers facing age-related changes in resources.

Based on a meta-analysis and review showing that the association between SOC and various work-related outcomes varied by the SOC components, Moghimi, Zacher [24] recommend that future research report the effects of overall SOC strategy use, as well as the separate SOC components, and their interactive effects on work outcomes. Therefore, it is important to distinguish between the three types of strategies in a SOC questionnaire. Thus, to test the validity of the questionnaire that we developed, we assessed whether it was able to distinguish between the three types of strategies: selection, optimisation, and compensation. 

So far, research has primarily investigated the use of SOC strategies at the individual level; however, it has been proposed that it may prove beneficial to broaden the scope of the SOC model beyond the individual [12,24,25,26,27]. For example, a recent study found that employees’ average use of SOC strategies buffered the negative association between their average age and company performance [28]. Particularly in a work context, the individual is likely to experience limited freedom to apply SOC strategies; for example, the individual employee may not have the freedom to select or prioritise work tasks when faced with a reduction in resources. However, the group and leadership levels may provide unique opportunities to apply SOC strategies for the benefit of all. For example, the group may provide unique opportunities for knowledge sharing and skill development (optimisation), or the immediate manager may expand the range of possible selection strategies. The importance of including the individual, group, and leadership levels when designing sustainable return-to-work schemes for employees has likewise been emphasised [29] because the various organisational levels may mutually reinforce each other. Explicitly exploring collective strategies used by the group of colleagues or the management may provide new and important insights into how workplaces can support long and healthy work lives for the employees. Therefore, the questionnaire we developed should be able to measure and distinguish between the employees’ perceptions of the use of SOC strategies at the three organisational levels (leadership, group, and individual). Thus, when testing the validity of the questionnaire, we assessed its capacity to distinguish between these three organisational levels.

Various questionnaires to measure the use of SOC strategies have been applied in SOC research, e.g., [2,30,31,32,33]; however, all existing SOC questionnaires only measure the individual’s use of SOC strategies. It is also a common issue for SOC scales to have low internal reliability (i.e., low Cronbach’s alpha-values) [8,9,10,14,19,34,35,36]. Furthermore, when measuring the use of SOC strategies at work, generic SOC questionnaires are commonly used, in which the respondent is asked to think of work when responding [26]. To better understand how SOC applies to the work setting, Moghimi and Scheibe [26] underline the need for questionnaires capturing work-specific manifestations of SOC. Abraham and Hanson [2] developed a questionnaire aimed specifically at work. However, this questionnaire has some limitations. First, because compensation is difficult to operationalise in a questionnaire, the authors came up with a solution to use “impression management” as compensation. While this certainly is an interesting compensation strategy, it may be regarded as a rather narrow concept; thus, many other useful compensation strategies will not be captured with this questionnaire. Second, all nine items operationalising optimisation reflected keeping up skills and abilities. While this indeed is a central aspect of optimisation, other important optimisation strategies will not be captured when using this questionnaire. Lastly, all items start with the words “I now…” indicating a permanent loss of resources often related to ageing or chronic illnesses. Thus, this questionnaire is less likely to capture SOC strategies as a response to temporary losses of resources or the use of optimisation throughout the career to maintain resources. These issues limit the broad application of the questionnaire as well as its use at all stages of the career. Müller and Weigl [10] developed an SOC in nursing scale capturing the manifestation of SOC strategies in the nursing profession. Despite including fewer items than the questionnaire described above, this scale captures a broader range of SOC strategies and reflects the continuous use of strategies; thus, to a greater extent, it captures the use of SOC strategies beyond those used as a response to age-related related loss of resources. 

Nevertheless, large-scale studies across different occupational groups would benefit from a work-specific SOC questionnaire that can be applied across various work settings. We therefore developed a questionnaire that should be applicable across different work settings and that captures SOC strategies that may be relevant at all stages of the career. We tested the validity and reliability of the questionnaire on data from two very different work settings.

In developing the questionnaire, we chose to take the employees’ perspective, which is a common approach when measuring the psychosocial work environment, e.g., [37]. A weakness of this approach is the potential to underestimate the use of strategies at the leadership level because the employees may not be aware of all the strategies being used at this level. However, as mentioned by von Bonsdorff and Zhou [28], directly asking management about HRM practices risks measuring their intentions rather than their actual use of the practices. This may also be the case when measuring the use of SOC strategies. An advantage of choosing the employees’ perspective is that the questionnaire can more easily be included in large-scale studies. 

Lastly, research has shown that the CEO and other endogenous factors at the organisational and managerial levels influence decisions made at other organisational levels in a given company [38]. Thus, it can be expected that factors such as the economic situation at the workplace, decisions made by top management, and managerial characteristics will affect not only the extent of SOC use but also to some extent determine which SOC strategies can and will be used at the individual, group, and leadership level. This calls for taking the organisational context into account when applying questionnaires measuring behaviour at the individual, group, and leadership levels. 

Thus, to better explore and understand the use of SOC strategies at work, and gain greater knowledge on the role of SOC for long and healthy work lives, there is a need to develop questionnaires that capture the manifestations of SOC across various work settings and measure the use of SOC strategies beyond the individual level. Such a questionnaire would also be useful for practitioners as a tool to provide input to discussions on the use of and need for SOC strategies at the various organisational levels, and thereby inform targeted interventions to support long and healthy work lives. 

To achieve this, we needed to develop a questionnaire that distinguishes between items addressing the three organisational levels of leadership (L), group (G), and individual (I)—LGI, as well as items operationalising the three types of strategies: selection (S), optimisation (O), and compensation (C) SOC. To test whether the questionnaire is applicable across different work settings, we needed to find this pattern of results across data representing different work settings.

The aim of the present study was to test the validity and reliability of our questionnaire in measuring the employees’ perceptions of the use of selection, optimisation, and compensation strategies at the leadership, group, and individual levels in two different work settings: nurses working in public hospitals and workers in the dairy industry. 

## 2. Methods

Design: We applied a cross-sectional survey design.

Population: To capture different work settings, we chose to include data from healthcare and industrial work in the study. More specifically, we included nurses working in public hospitals in Denmark and Danish dairy workers. Work at a dairy plant is, to a large extent, characterised by physically demanding work tasks and unskilled work. Nursing is skilled work and also extensively includes emotional and cognitive demands, as well as being physically demanding.

## 3. Materials

### 3.1. The SOC Questionnaire

Because the questionnaire has not been described elsewhere, we include information on the development of our questionnaire in its description below.

#### 3.1.1. The Development of the Questionnaire Items for the SOC Scales

The development of the items in the questionnaire was based on existing SOC questionnaires measuring SOC at work [2,10,31], SOC theory, and qualitative interviews conducted at an earlier stage of the present project. In the earlier stage of the project, we conducted 19 individual and four focus group interviews, two focus groups with nurses, and two with dairy workers. Of the 19 individual interviews, 10 were with dairy workers, two of whom were managers, and nine with nurses, one of whom one had an HR position and one a manager position. The qualitative interviews explored, among other things, the manifestation of SOC strategies at all the organisational levels at the two types of workplaces selected for the study, and thus provided inspiration for items capturing SOC at the various organisational levels. The interviews were transcribed, coded, and analysed using thematic content analysis. Selected findings from the interviews are presented under the description of the development of the items in the questionnaire below, but the elaborate results of the qualitative interviews are the focus of a separate article.

To refine and validate the formulation of the individual items and exclude malfunctioning items, we conducted eight individual cognitive interviews [39] (four with dairy workers and four with nurses). The cognitive interviews were conducted in loops, which allowed revision of the items after one or two cognitive interviews, and then conducting one or two more cognitive interviews, repeating the process of revision following interviews. The cognitive interviews also allowed us to check that the items we had constructed based on the theoretical input were meaningful to the informants and captured strategies that they actually used in practice. After this process, we had a questionnaire with 29 items (see Table 1), which we used in the cross-sectional survey to further validate the questionnaire.

#### 3.1.2. Theoretical Considerations When Operationalising SOC Behaviour as Questionnaire Items

In the literature, a distinction is made between loss-based and elective selection, and the original SOC questionnaire includes this distinction [31]. However, the two SOC questionnaires developed for workplaces do not distinguish between the two types of selection [2,10]. It also posed a challenge to make this distinction when operationalising selection beyond the individual level. In the qualitative interviews, we encountered manifestations of both loss-based selection (e.g., an immediate manager relieved an employee from a specific workstation because she had pain in her collarbone) and elective selection (e.g., to reduce the physical strain, the immediate manager assigned an employee to a teaching task, taking into account that she liked teaching). Nevertheless, when operationalising the strategies in the questionnaire, it was difficult to distinguish between the two types of selection without the statements becoming too long and job-specific, compromising the applicability of the items across different work settings. We therefore did not distinguish between the two types of selection in the questionnaire.

SOC strategies are used as a response to limited resources and to maintain resources [30]. As mentioned in the introduction, we aimed to capture SOC strategies not only related to ageing, but also as a response to temporary limitations in resources caused by other factors throughout the entire career. To achieve this, we introduced the expression “to be under pressure” to capture situations where resources are lacking and the use of SOC strategies is potentially beneficial (Item 1, 10, 19, 20, 21 in Table 1). We also introduced the expressions “unable to perform some work tasks” and “if a work task puts too much strain on” as these situations are likely to be caused by limited resources for various reasons, and again, the use of SOC strategies is potentially beneficial (Item 2, 3, 11, 22 in Table 1). 

When operationalising optimisation, Abraham and Hanson [2] focus on retaining skills and knowledge relevant for work. Müller and Weigl [10] introduced the concept of ergonomics when operationalising optimisation in their SOC in nursing questionnaire. They also introduced the use of technical assistive devices to complete heavy physical tasks as compensation. We chose to distinguish between technical assistive devices used preventatively to generally reduce strain and categorised this as optimisation in line with using ergonomically correct working positions. Special or personal technical assistive devices that make it possible to complete a work task despite a reduction in functional ability were categorised as compensation.

In some cases, the same behaviour can reflect both compensation and optimisation. To distinguish between using assistive technical devices as optimisation and as compensation and to capture compensation more broadly, we wanted the respondent to think of situations where employees experience difficulties completing some of their work tasks and thus have to turn to alternative means or external resources to complete the tasks. The use of alternative means and external resources is the essence of compensation [31]. We therefore added the following introduction text to this group of items: “Many people experience temporary or permanent discomfort/troubles that can make it difficult to complete some work tasks. This can be in connection with, for example, pain, illness, injury, stress, handicap, or getting older or worn down”. Depending on the organisational level the statements are addressing, this sentence was followed by: “The following statements concern the cooperation with the immediate manager when handling an employee having difficulties in performing some work tasks/the cooperation in the group regarding handling when a colleague has difficulties performing some job tasks/how you handle or handled the work despite this”.

#### 3.1.3. Operationalisation of SOC at the Organisational Level

Acknowledging that behaviour at the upper management level affects behaviour at the lower levels of an organisation [38], we originally wanted to include the organisational level in the questionnaire. In the qualitative interviews, we encountered manifestations of selection, optimisation, and compensation at the organisational level. However, it turned out to be very difficult to distinguish between the three types of strategies when attempting to operationalise them in the questionnaire. As a consequence, we decided to abandon the aim to measure SOC at the organisational level and only included SOC at the leadership, group, and individual levels in the questionnaire.

#### 3.1.4. Operationalisation of SOC at the Leadership Level

In the qualitative interviews, selection at the leadership level primarily manifested itself as the immediate manager finding alternative tasks for employees. It may be possible for a manager to find alternative tasks for an employee for a limited period of time, but it will often be much more difficult on a permanent basis. Therefore, when operationalising selection at the leadership level, we included a statement addressing both short-term and long-term change of tasks (Item 2 & 3 in Table 1).

Otherwise, as presented in Table 1, the operationalisation of optimisation and compensation at this level very much reflect the immediate manager providing direct support for the employees’ use of SOC strategies at the individual level.

#### 3.1.5. Operationalisation of SOC at the Group Level

From the qualitative interviews, we learned that selection at the group level mainly manifested itself as group members swapping work tasks around so that a colleague could avoid tasks that either strained him or her or the person was not able to perform. Therefore, the statements capturing selection at the group level primarily reflect the exchange of work tasks in the group.

Müller and Weigl [10] included “asking for help to complete heavy physically demanding tasks” as compensation in their questionnaire. Again, as with the use of technical assistive devices, we chose to distinguish between group members generally helping each other with straining tasks as a means to prevent strain, and thereby maintain resources, and categorised this as optimisation, while we categorised colleagues helping a colleague who is not able to complete a task due to a functional problem as compensation because it reflects the use of alternative means or external resources to be able to complete the task [31].

The operationalisation of SOC at the group level is a mixture of shared strategies in the group (e.g., items 10 & 15 in Table 1) and the group members providing direct support for each other’s individual use of SOC strategies (e.g., items 11 & 14 in Table 1).

Furthermore, we applied the method of reference shift consensus [40], where the respondent is required to respond from a group perspective rather than their own individual experiences. This approach was used to capture group-level constructs based on individual responses.

#### 3.1.6. Operationalisation of SOC at the Individual Level

In general, the statements capturing selection at the individual level reflect the existing SOC questionnaires [2,10], such as completing one task at a time and reducing the number of work tasks. Given that it is not always possible to just abandon a work task, we included the item “If a work task puts too much strain on me, I ask to get removed from the task” to operationalise selection. Although this may not always result in the employee being exempted from the task, it reflects a strategy to deal with limited resources by attempting to deselect this work task.

In the qualitative interviews, there were also quite a few examples of strategies for recovery, for example, to ask for an extra day off after night shifts or relax on the sofa when coming home from work to recover after a straining workday. It could be argued that strategies to recover may be regarded as a way to allocate or maintain resources to perform the work tasks or reach the goal, and thus considered an optimisation strategy. To capture recovery without being too specific, we included the item “I take the breaks I need” when operationalising optimisation.

Because we decided to focus on SOC strategies applied while working or at work, we did not include doing exercise to stay fit, even though it came up in the qualitative interviews and is included in the SOC in nursing questionnaire [10].

Compensation strategies tend to be very specific. In the qualitative interviews, one interviewee explained how she used the other side of her hand to lift things when she had pain, and another interviewee reported how he would rise from his chair to put things on the shelves above his head because it was painful to lift the arms. To capture these types of strategies, where alternative means are used to complete the task, we included the item “If I have troubles causing difficulties performing some of my work tasks, I try to find other ways of performing them”. The results of the cognitive interviews indicated that this worked well; the examples the respondents came up with fitted well with the type of strategies we aimed to capture.

As mentioned earlier, to capture compensation, we included the introduction text, “Many people experience temporary or permanent discomfort/troubles that can make it difficult to complete some work tasks (…..)”. However, to avoid annoying respondents who have not experienced troubles causing difficulties performing work tasks, we included the following screening question: “Have you experienced troubles that made it difficult for you to manage some of your work tasks?”. It had response options on a six-point Likert-type scale ranging from 1 = “not at all” to 6 = “to a very large extent”. Persons who had responded “not at all” to this question would not be presented with the three statements regarding handling these challenges (Items 27, 28, & 29 in Table 1). This meant that these respondents would not recieve a compensation score at the individual level. The results from the cross-sectional questionnaire survey revealed that 16% of the dairy workers and 24% of the nurses responded “not at all” to this screening question.

#### 3.1.7. Response Options for the Items

The original SOC questionnaire and its short version use forced choice responses where there for each SOC item is a corresponding item not reflecting SOC behaviour [33]. Later, Zacher and Frese [41] developed a revised version of the short SOC questionnaire using a five-point Likert-type scale as response option. The work-specific SOC questionnaires similarly use a Likert-type scale as response options [2,10]. To minimise the amount of text, we therefore also applied a single item option with a five-point Likert-type scale ranging from 1 = “not at all/to a very low extent” to 5 = “to a very large extent”. In addition, some items had a response option indicating that the strategy was not relevant for the respondent (Item 1, 4, 5, 8, 9, 13, 22, 27, 28, and 29 in Table 1).

### 3.2. Additional Scales Included in the Cross-Sectional Questionnaire Survey

We wanted to test the criterion validity of the SOC scales. As mentioned in the introduction, research has found a positive association between SOC at the individual level and wellbeing [11,12,13,14] and job satisfaction [20,21]. We therefore expected the SOC scales in our questionnaire to be associated with these two outcomes and included measures of wellbeing and job satisfaction in the survey.

#### 3.2.1. Wellbeing

Wellbeing was measured with the five-item WHO-5 wellbeing scale [42] in which participants responded on a six-point Likert-type scale ranging from “all of the time” to “none of the time”. Responses were added into a scale and rescaled from 0–100 in the analyses (Cronbach’s alpha: data from nurses: 0.88 and data from dairy workers 0.85). 

#### 3.2.2. Job Satisfaction

Job satisfaction was measured with the item “how satisfied are you with your job as a whole, taking everything into account” from the Danish Working Environment and Health questionnaire [43], where participants responded on a five-point Likert-type scale ranging from “very dissatisfied” to “very satisfied”. In the analyses, the scale was also rescaled from 0–100.

### 3.3. Participants and Procedure in the Cross-Sectional Questionnaire Survey

For practical reasons, the recruitment of participants and the procedure for conducting the survey differed for the nurses and dairy workers.

Nurses: The nurses were recruited through the Danish Nurses Organisation. Inclusion criteria were nurses working at public hospitals and currently employed. A random sample of 2000 members of the organisation, who, according to the database, fulfilled these criteria, was selected for the study. Of these, 1966 had a valid e-mail address and received an e-mail with an invitation to participate in the questionnaire survey and a link to the online questionnaire. Data were collected during April 2018. To ensure the highest possible response rate, reminders were sent out twice during these four weeks. We received responses from 850 persons, corresponding to a response rate of 43%. Out of these, 65 were excluded for the following reasons: eight were temporarily unemployed, 25 were not working as nurses, 16 were not employed at public hospitals, and 16 had only responded to the first two screening questions. Thus, the total sample consisted of 785 nurses employed at public hospitals.

The nurses had a mean age of 47 years (SD 12) ranging from 23 to 70 years; 85% were women, 4% were men, and 11% did not report their gender. In the sample, 29% worked at medical wards, 26% at surgical wards, 18% at psychiatric wards, and 33% at “other wards,” including emergency, intensive care units, X-ray, hospice, and administration.

Dairy workers: Four dairies were recruited for the study through the Danish Dairy Cooperative Forum. Letters for all employees with invitations to participate in the study and a code to access the online questionnaire were sent to the contact person in each dairy, who then ensured they were distributed to the employees. Data were collected in May 2018. During these four weeks, to ensure the highest possible response rate, reminders with information about the current response rate were sent to the contact persons, who then encouraged team leaders to encourage their employees to fill in the questionnaire. Reminders were sent three to five times depending on when the contact person thought the maximum possible participation had been reached. Across the four dairies, a total of 490 employees were invited to participate, out of which 244 responded (50%). Their mean age was 47 years (SD 10), ranging from 21 to 65 years; 30% were women, 65% were men, and 5% did not report their gender.

#### 3.3.1. Ethics

Data collection and handling was approved by the internal board at the National Research Centre for the Working Environment on 25 October 2017, with approval number “rev: 01.12.2016”.

Prior to accessing the link to the electronic questionnaire, the participants received information about the purpose of the study, the content of the questionnaire, and the handling of the data. By continuing to the electronic questionnaire, the participants provided informed consent to participate in the study. 

#### 3.3.2. Analyses

We developed a questionnaire that should be able to distinguish between items at the three organisational levels leadership (L), group (G), and individual (I)—LGI, *as well as* items operationalising the three types of strategies selection (S), optimisation (O), and compensation (C)—SOC. Therefore, to test the construct validity of the questionnaire, we investigated the factor structure of the questionnaire in confirmative factor analyses (CFA), testing four models: (1) A 1-factor model—all items load on the same factor; (2) A 3-factor model, with one factor for each of the organisational levels (Leadership, Group, Individual); (3) A 3-factor model, with one factor for each of the three types of SOC strategies; and (4) A 9-factor model with one factor for each organisational level and further divided into the three types of strategy (LS, LO, LC, GS, GO, GC, IS, IO, and IC). To achieve a satisfactory model fit, the SRMR needed to be less than 0.09, the RMSEA less than 0.08, and the CFI 0.95 or more [44]. We used the sequential chi-square difference test (SCDT) [45] to compare factor models; a value greater than 1.96 indicates a significant improvement of the model fit. We compared whether the two three-factor models had a significantly superior fit than the 1-factor model, and whether the nine-factor model had a significantly superior fit to the two 3-factor models respectively.

To test the internal consistency reliability of the nine subscales, we calculated Cronbach’s alpha for all subscales in the questionnaire. Lastly, to evaluate the criterion validity of the subscales, we tested the univariate associations between the nine scales and the two outcomes, “wellbeing” and “job satisfaction,” with linear regression analysis controlling for age and gender. CFA analyses were run in M-Plus 7, and Cronbach’s alpha and regression analyses in SPSS statistics 25. All analyses were run separately on the data from nurses and dairy workers respectively.

## 4. Results

### 4.1. Assessment of the Construct Validity of the Questionnaire and the Internal Consistency Reliability of the Subscales

In data from the nurses, the results from the Sequential chi-square difference test (SCDT) [45] showed that the two three-factor models both had a significantly better model fit than the one-factor model (three factors (LGI): ΔꞳ^2^/Δdf = 494.3 & three factors (SOC): ΔꞳ^2^/Δdf = 6.0). These findings indicate that the data fit better when the questionnaire differentiated between the three organisational levels (LGI) or the three SOC strategies. A comparison of the nine-factor model with the two 3-factor models showed that the nine-factor model had a significantly better model fit than both of the three-factor models (three factor (LGI): ΔꞳ^2^/Δdf = 7.3 & three factor (SOC: ΔꞳ^2^/Δdf = 51.7). Thus, the results showed that the data fit best when the questionnaire differentiated between the three organisational levels (LGI) and the three types of strategies (SOC). Furthermore, the model fit indices showed that the nine-factor model achieved a satisfactory model fit (results are shown in Table 2). Together, the results support the factor structure of the questionnaire that we aimed to achieve.

We then calculated Cronbach’s alpha for the respective subscales. The majority of the scales had acceptable internal consistency reliability. However, the scale for optimisation at the group level (GO) and the three scales at the individual level (IS, IO, IC) had alpha values below 0.70, which is considered the generally accepted threshold for satisfactory internal reliability. The Cronbach’s alpha values for the respective subscales are shown in Table 3.

In data from dairy workers, the results from the SCDT test showed that both of the three-factor models had significantly better fit than the one-factor model (three factor (LGI): Δ X^2^/Δdf = 136.0 & thee-factor (SOC): Δ X^2^/Δdf = 10.7). These results indicated that the data fitted better when the questionnaire differentiated between the three organisational levels (LGI) or the three SOC strategies in this dataset as well. As in the data from the nurses, the nine-factor model had a significantly better fit than both of the three-factor models (three factor (LGI): ΔX^2^/Δdf = 3.7 & three-factor (SOC): ΔX^2^/Δdf = 15.1). Thus, the results showed that the data fitted best when the questionnaire differentiated between the three organisational levels (LGI) and the three types of strategies (SOC) in the data from the dairy workers as well. Lastly, the nine-factor model almost achieved a satisfactory model fit (results are shown in Table 2). Overall, the results support the factor structure of the questionnaire that we aimed to achieve in this dataset.

We then calculated Cronbach’s alpha for the respective subscales, and the results showed the same pattern as in the data from the nurses. The majority of the scales had acceptable internal consistency reliability, but the optimisation scale at the group level (GO) and the three scales at the individual level (IS, IO, IC) had alpha values below the generally accepted threshold for satisfactory internal reliability (0.70). The Cronbach’s alpha values for the respective subscales are shown in Table 3.

Lastly, we calculated Pearson’s correlation between the nine subscales in the questionnaire in both datasets. The results showed the same overall pattern in both datasets. Within the organisational level, the subscales at the individual level had the weakest mutual correlations, while the mutual correlations between the subscales were strong at the group and leadership levels. Looking at correlations across the organisational levels, the majority were weak or moderate (the correlation matrices are presented in the Appendix A).

### 4.2. Assessment of the Criterion Validity

The results showed that all nine scales (LS, LO, LC, GS, GO, GC, IS, IO, OC) were significantly associated with wellbeing in the data from the nurses. In data from the dairy workers, all scales except selection at the individual level (IS) were significantly associated with wellbeing (see Table 4). These findings provide support for the criterion validity of the scales.

Regarding job satisfaction, the results showed that all scales except selection at the individual level (IS) were significantly associated with job satisfaction in the data from the nurses. In data from the dairy workers, only five of the nine scales were significantly associated with job satisfaction, with the exceptions being LS, LC, GK, and IC (See Table 4). Thus, these results provide partial support for the criterion validity of the scales.

## 5. Discussion

We developed a questionnaire to measure employees’ perception of the use of SOC strategies at the leadership, group, and individual levels, which would be applicable across various types of work settings. The aim of the present study was to test the validity and reliability of the questionnaire.

To test the construct validity of the questionnaire, we tested the factor structure of the questionnaire with confirmative factor analyses aiming to achieve a nine-factor model. The results showed that the nine-factor model had the best fit, of the four models tested, in both datasets. Furthermore, we managed to achieve a satisfactory model fit in the data from the nurses and an almost satisfactory model fit in the data from the dairy workers. The three subscales at the individual level, as well as optimisation at the group level, had low internal consistency reliability, while the rest of the subscales had acceptable reliability in both datasets. Furthermore, the results showed that the majority of the subscales were moderately correlated in both datasets. Finally, we assessed the criterion validity of the scales. Results showed that all scales in the data from nurses and all scales except one (IS) in data from the dairy workers were significantly associated with wellbeing, providing support for the criterion validity of the scales. However, while all scales except one (IS) were significantly associated with job satisfaction in data from the nurses, only five of the nine scales were significantly associated with job satisfaction in data from the dairy workers. These results, thus, only provide partial support for the criterion validity of the scales. 

An assessment of the construct validity of the questionnaire revealed that the nine-factor model had the best fit of the four models tested in both datasets, and the nine-factor model achieved satisfactory model fit in one dataset and almost satisfactory model fit in the other dataset. These findings indicate that the questionnaire is able to distinguish between the three types of strategies (selection, optimisation, compensation) and the three organisational levels (leadership, group, individual). We thus succeeded in developing a questionnaire that measures employees’ perceptions of the use of SOC strategies beyond the individual level. This questionnaire is an important tool for research into the associations between SOC strategies at the individual, group, and leadership levels, and indicators for long and healthy work lives. The questionnaire may also be valuable for practitioners because it provides an indication of the extent of SOC use at the three organisational levels. In developing targeted interventions to support long and healthy work lives, the questionnaire can help draw attention to aspects of the organisation that may benefit from implementation efforts to enhance the use of SOC strategies, supporting long and healthy work lives. Nevertheless, continued refinement of the questionnaire may be needed.

We aimed to develop a questionnaire that was applicable across work settings. The results showed the same overall structure in the data from the two work settings included in the study. These findings indicate that the questionnaire may be used across different work settings. Nevertheless, while we managed to achieve a satisfactory model fit in the data from the nurses, the data from the dairy workers did not quite achieve a satisfactory model fit. These results indicate that the questionnaire may be a better operationalisation of SOC at the three organisational levels in some work settings than others. Further, many types of work settings were not represented in our study, such as knowledge work. We therefore cannot make any definite conclusions as to whether the questionnaire is applicable across occupational groups. Thus, further validation across a broader range of occupational groups is needed. Müller and Weigl [10] argue that the manifestation of SOC strategies depends on the work context and thus advocate for the development of job-specific SOC questionnaires. Future research will help determine whether efforts may be better spent in adjusting the questionnaire to specific job groups and validating it within these job groups, rather than adhering to the aim of developing a questionnaire that is valid across job groups. However, having job-specific SOC questionnaires will be a disadvantage when conducting large-scale studies exploring the perceived use of SOC across occupational groups, because various versions of the SOC questionnaire would have to be distributed in such a case.

The results showed that many of the subscales had moderate mutual correlations, which is to be expected. For example, the perceived use of the strategies at one organisational level is likely to influence the use of the strategies at other organisational levels. If the immediate manager encourages staff to use technical assistive devices, chances are that the individual employee is more likely to use these devices. In the cognitive interviews, we also learned that the use of a strategy at one level led to the strategy not being used at other levels. For example, the immediate manager did not help in prioritising work tasks, because the employees managed this on their own at the group level. Thus, even though selection, optimisation, and compensation at the different organisational levels can be regarded as theoretically separate entities, in practice, they are inter-related. This supports Moghimi and Zacher’s [24] recommendation to analyse the interactive effects of SOC components on work outcomes, and could be expanded to include interactive effects across organisational levels as well. It also has implications for practitioners. Practitioners who use the questionnaire in the workplace should take a holistic approach when interpreting the results. They should not only focus on having a high score at all organisational levels but also explore whether the need for strategies is fulfilled at the most appropriate level at that specific workplace. 

Regarding the reliability of the questionnaire, the results showed that the subscales at the leadership and group levels (except optimisation at the group level) had acceptable Cronbach’s alpha values, while the subscales at the individual level tended to have low alpha values in both datasets. In the literature, it is not unusual for researchers to report low alpha values for the SOC subscales, which are all at the individual level [8,9,10,14,19,34,35,36]. Many of these authors highlight the argument that the scales cannot be expected to have high internal consistency because SOC captures a broad phenomenon. Our results indicate that this is less of an issue at the other organisational levels, which makes sense. At the group and leadership levels, one would, to a larger extent, expect the use of the respective strategies to be related because different employees and colleagues may have different needs; thus, a broader repertoire of strategies will be relevant. However, at the individual level, the use of one strategy cannot necessarily be expected to imply the use of another strategy. It may even be that the use of one strategy makes a person less likely to use other strategies. For example, an individual may ask for help (compensation) and thus have less need to use other compensation strategies, such as individual assistive technical devices, and therefore would report limited or no use of the other compensation strategies. The low alpha values often reported for the SOC scales and found in this study at the individual level call for consideration of whether they are best treated as scales or as single items.

Finally, we assessed the criterion validity of the scales by analysing the association between the nine scales and the two outcomes, wellbeing, and job satisfaction, which have been found to be associated with SOC at the individual level in the literature. All scales were significantly associated with wellbeing except selection at the individual level (IS) in data from the dairy workers. The IS scale also had the weakest association in the data from the nurses, and was the only scale in the data from the nurses that was not significantly associated with job satisfaction. This could indicate a weakness in this scale. In the data from dairy workers, only five of the nine scales were significantly associated with job satisfaction, and thus only provided partial support for the criterion validity of the scales. The sample size was rather low in the data from dairy workers, limiting the statistical power, which may be part of the explanation for the lack of significant associations. Overall, while results from analyses with wellbeing as an outcome provide support for the criterion validity of the SOC scales, in data from the dairy workers, the results only provide partial support for the criterion validity when job satisfaction was used as outcome. Therefore, further research exploring the criterion validity of the scales is recommended. 

The use of SOC at the group and leadership levels can be expected to be influenced by social relations in the workplace. We therefore recommend that future research explore how psychosocial aspects, such as social capital within teams and in the relationship with the immediate manager [46], and psychological safety [47] interact with and influence the perceived use of SOC at the group and leadership level. It would also be relevant to explore how various management styles are associated with and interact with perceptions of the use of SOC at the leadership level. As mentioned in the introduction, previous research has shown that managers’ behaviour is influenced by various endogenous factors in the organisation [38]. The use of SOC strategies at the leadership, group, and individual levels can likewise be expected to be influenced by factors at the top organisational level, such as the economic situation, policies, and top management attitudes towards the retention of staff and so forth. Therefore, in applying the questionnaire across workplaces, we recommend that organisational factors be taken into account when analysing the data and drawing conclusions from the results.

In general, further refinement and validation of the questionnaire are encouraged, as it still has some weaknesses. We only included the perspective of the individual employees on the use of strategies at the various organisational levels, which may introduce a bias. It would be interesting in future research to expand the SOC questionnaire to include the perspective of, for example, management on the use of SOC strategies and to compare this with the responses of the employees. With the current version of the questionnaire, we cannot rule out the possibility that SOC strategies are being used at the leadership level, of which the employees may be unaware. Such an expansion of the questionnaire would, however, make it more challenging to apply it to large-scale studies because different respondents would have to answer different aspects of the questionnaire. 

The operationalisation of compensation has challenged researchers in the field [2]. We aimed to capture compensation at the individual level by including items addressing strategies applied when the respondents had troubles causing difficulties in performing some of their work tasks. To avoid annoying participants who had not experienced this, we introduced a screening question, “Have you experienced troubles that made it difficult for you to manage some of your work tasks?” before the three items that measured compensation at the individual level. However, the proportion of respondents excluded due to this screening question was much larger than expected (16% of the dairy workers and 24% of the nurses), leading to a high number of missing values and exposing a weakness in the questionnaire. One solution may be to broaden the range of examples that may cause these troubles so that more respondents recognise these as relevant for them. The current wording is “Many people experience temporary or permanent discomfort/troubles that can make it difficult to complete some work tasks. This can be in connection with, for example, pain, illness, injury, stress, handicap, or getting older or worn down”. Perhaps one could add “in connection with pregnancy” or “in connection with difficult life circumstances,” which would also make the questionnaire even better at capturing compensation strategies applied as a response to limited resources throughout the entire career.

A response option specifying that one has not experienced this type of challenge could be added rather than retaining the screening question. We expect that the respondents would be able to relate to at least some of the three items if introduced to them. To further reduce the number of missing values, we do, nevertheless, encourage future research to attempt to reduce the number of questionnaire items that include response options indicating that the strategy is not relevant.

### Strengths and Weaknesses of the Study

A major strength of this study is that it is the first study to develop a questionnaire to measure employees’ perceptions of the use of SOC strategies beyond the individual level. The study thereby contributes a tool to measure perceived SOC behaviour beyond the individual level, which has so far been lacking in research and practice. Great effort was put into the development of the items in the questionnaire, which were based on SOC theory, qualitative interviews, and existing SOC questionnaires, as well as cognitive interviews to validate the items.

Another strength of the study is that we developed and validated the questionnaire using data from two different work settings (health care and industrial work). Our theoretical assumption that the questionnaire differentiates between the three types of SOC strategies and the organisational levels was confirmed in both datasets, and the overall structure of results was the same in the two datasets, indicating a fairly broad application of the questionnaire across work settings. Nevertheless, there are still many types of workplaces that were not represented, such as knowledge work; thus, further validation and adjustment of the questionnaire across a larger range of occupational groups is needed.

Regarding the assessment of criterion validity, the analyses were based on self-reported cross-sectional data, which poses the risk of common method bias [48], with the potential of inflating the strength of the observed associations.

Furthermore, we achieved response rates of 43% and 50% for nurses and dairy workers, respectively. Even though the response rates were quite good for survey data, it was not possible to obtain information about people who had not responded; thus, we were not able to evaluate how representative our sample was. The sample size of dairy workers was fairly small, particularly for the CFA and regression analyses, where *n* was reduced substantially due to missing values.

## 6. Conclusions

This study is the first to develop a questionnaire measuring employees’ perceptions of the use of SOC strategies at work beyond the individual level. Results from the test of the validity and reliability of the questionnaire largely supported the construct and criterion validity of the questionnaire as well as the internal consistency reliability of the subscales. The study thus contributes an important tool for research into the role of SOC strategies at the individual, group, and leadership levels for long and healthy working lives. It may also be used by practitioners as a tool to provide input when developing targeted interventions to support long and healthy working lives. Nevertheless, research further validating the questionnaire, particularly across a broader range of occupational groups, is needed.

## Figures and Tables

**Table 1:** The 29 SOC items included in the questionnaire.

	**Leadership level**
	***Selection***
1	My immediate manager helps prioritising work tasks if an employee is under a lot of pressure.
2	If an employee is unable to perform some work tasks for a limited period of time, my immediate manager finds other types of work tasks for the employee for that period of time.
3	If an employee is permanently unable to perform some work tasks, my immediate manager will find other types of work tasks for the employee on a permanent basis.
	***Optimisation***
4	My immediate manager encourages the employees to use the available technical assistive devices to ensure their safety and health.
5	My immediate manager encourages the employees to use ergonomically correct working postures.
6	My immediate manager makes an effort in fulfilling employees’ wishes about learning new things that are important for the work.
	***Compensation***
7	If an employee needs particular technical assistive devices to perform his/her work tasks, my immediate manager will help acquire them.
8	If an employee has difficulties in performing a work task, my immediate manager will talk with the employee about other ways to perform the work task.
9	If an employee has difficulties performing some of his/her work tasks, my immediate manager will arrange for someone to help the employee with the work tasks.
	**Group level**
	***Selection***
10	If we are under pressure, we jointly prioritise the work tasks in the group.
11	If someone in the group feels that a work task puts too much strain on him/her, we exchange some of the work tasks.
12	If someone in the group has troubles causing them difficulties in performing some of their work tasks, we exchange some of the work tasks.
	***Optimisation***
13	In my group, we usually help each other with heavy/demanding tasks.
14	In my group, we usually encourage each other to use the technical assistive devices that are available to ensure safety and health.
15	In my group, we share new work related knowledge with each other.
	***Compensation***
16	If someone in the group has troubles causing difficulties in performing some of his/her work tasks, a colleague will help carrying out the tasks.
17	If someone in the group has troubles causing difficulties in performing some of his/her work tasks, we discuss if there are other ways to perform the tasks.
18	If someone in the group has troubles causing difficulties in performing some of his/her work tasks, we discuss if there are any particular individual technical assistive devices that may help him/her.
	**Individual level**
	***Selection***
19	If I feel under pressure, I deselect less important tasks.
20	If I feel under pressure, I complete one task before I move on to the next.
21	If I feel under pressure, I try to reduce the number of work tasks.
22	If a work task puts too much strain on me, I ask to get removed from the task.
	***Optimisation***
23	I usually make use of the technical assistive devices available to ensure safety and health.
24	I usually make sure to use ergonomically correct working postures.
25	I take the breaks I need.
26	I put effort into learning new things that are important for my work.
	***Compensation***
27	If I have troubles causing difficulties in performing some of my work tasks, I ask my colleagues for help.
28	If I have troubles causing difficulties in performing some of my work tasks, I try to find other ways of performing them.
29	If I have troubles causing difficulties in performing some of my work tasks, I ask for technical assistive devices

**Table 2:** Results from CFA analyses of model fit in the two datasets.

	SRMR	RMSEA	CFI	X^2^ (df)
**Data from nurses** (*n* = 310)				
one factor	0.125	0.136	0.76	2535 (377)
three factors (L, G, I) *	0.079	0.076	0.93	1052 (374)
three factors (S, O, C) **	0.123	0.129	0.78	2517 (374)
nine factors (LS, LO, LC, GS, GO, GC, IS, IO, IC) ***	0.067	0.067	0.95	812 (341)
**Data from dairy workers** (*n* = 103)				
one factor	0.152	0.136	0.78	1096 (377)
three factors (L, G, I) *	0.110	0.090	0.90	688 (374)
three factors (S, O, C) **	0.152	0.134	0.79	1064 (374)
nine factors (LS, LO, LC, GS, GO, GC, IS, IO, IC) ***	0.095	0.080	0.93	566 (341)

* three factors: the organisational levels (leadership, group; individual), ** three factors: the SOC strategies (Selection, Optimisation, Compensation), *** nine factors: The organisational levels and further divided by SOC strategies.

**Table 3:** Cronbach’s alpha for the subscales in the questionnaire.

Subscale	Number of Items	α Data from Nurses (*n* = 344–364)	α Data from Dairy Workers (*n* = 128–143)
*Leadership level*			
Selection (LS)	3	0.75	0.76
Optimisation (LO)	3	0.77	0.71
Compensation (LC)	3	0.80	0.86
*Group level*			
Selection (GS)	3	0.80	0.82
Optimisation (GO)	3	0.56	0.61
Compensation (GC)	3	0.83	0.82
*Individual level*			
Selection (IS)	4	0.54	0.57
Optimisation (IO)	4	0.52	0.65
Compensation (IC)	3	0.57	0.55

**Table 4:** Results from the regression analyses with wellbeing and job satisfaction respectively as outcome variable divided by the two datasets.

Wellbeing							Job Satisfaction					
	Data from Nurses (*n* = 348–359)			Data from Dairy Workers (*n* = 127–134)			Data from Nurses (*n* = 348–359)			Data from Dairy Workers (*n* = 127–134)		
	Std. β	SE	*p*	Std. β	SE	*p*	Std. β	SE	*p*	Std. β	SE	*p*
LS	0.288	0.045	0.000	0.214	0.065	0.010	0.418	0.055	0.000	0.124	0.100	0.149
LO	0.286	0.044	0.000	0.226	0.061	0.007	0.378	0.055	0.000	0.226	0.107	0.009
LC	0.301	0.046	0.000	0.329	0.058	0.000	0.409	0.056	0.000	0.137	0.109	0.112
GS	0.343	0.052	0.000	0.351	0.063	0.000	0.399	0.065	0.000	0.293	0.115	0.001
GO	0.368	0.058	0.000	0.324	0.079	0.000	0.355	0.075	0.000	0.214	0.146	0.016
GC	0.334	0.050	0.000	0.328	0.075	0.000	0.396	0.062	0.000	0.124	0.140	0.189
IS	0.137	0.071	0.009	0.088	0.075	0.292	0.067	0.092	0.208	0.211	0.130	0.013
IO	0.365	0.075	0.000	0.409	0.079	0.000	0.331	0.097	0.000	0.168	0.152	0.049
IC	0.257	0.054	0.000	0.330	0.062	0.000	0.179	0.071	0.001	0.050	0.116	0.562

All associations are controlled for gender and age.

## Data Availability

Due to the Danish data protection act, data cannot be made public available. Researchers interested in access to the data is encouraged to contact the corresponding author.

## Appendix A. Pearson’s Correlation between the Subscales of the Questionnaire

**Table A1:** Data from nurses.

	LS	LO	LC	GS	GO	GC	IS	IO	IC
LS	1								
LO	0.64	1							
LC	0.83	0.61	1						
GS	0.37	0.32	0.38	1					
GO	0.35	0.39	0.35	0.65	1				
GC	0.42	0.35	0.45	0.77	0.59	1			
IS	0.23	0.16	0.21	0.26	0.24	0.29	1		
IO	0.28	0.31	0.28	0.41	0.52	0.44	0.26	1	
IC	0.19	0.24	0.20	0.41	0.35	0.40	0.43	0.40	1

*n* = 351–364, all correlations are significant at the *p* < 0.01 level.

**Table A2:** Data from dairy workers.

	LS	LO	LC	GS	GO	GC	IS	IO	IC
LS	1								
LO	0.66 **	1							
LC	0.79 **	0.69 **	1						
GS	0.34 **	0.21 *	0.39 **	1					
GO	0.37 **	0.37 **	0.47 **	0.71 **	1				
GC	0.31 **	0.27 **	0.35 **	0.69 **	0.63 **	1			
IS	0.27 **	0.11	0.27 **	0.35 **	0.30 **	0.35 **	1		
IO	0.24 **	0.45 **	0.42 **	0.35 **	0.52 **	0.37 **	0.23 **	1	
IC	0.25 **	0.20 *	0.30 **	0.50 **	0.57 **	0.53 **	0.45 **	0.40 **	1

*n* = 135–143, * *p* < 0.05, ** *p* < 0.01.
